# Clinical Relevance of Pathological Diagnosis of Hirschsprung’s Disease with Acetylcholine-Esterase Histochemistry or Calretinin Immunohistochemistry

**DOI:** 10.3390/children11040428

**Published:** 2024-04-03

**Authors:** Philipp Romero, Astrid Burger, Erica Wennberg, Stefanie Schmitteckert, Stefan Holland-Cunz, Constantin Schwab, Patrick Günther

**Affiliations:** Department of Surgery, Division of Pediatric Surgery, University of Heidelberg, Im Neuenheimer Feld 430, 69120 Heidelberg, Germany; astrid.burger@med.uni-heidelberg.de (A.B.); erica.wennberg@mail.utoronto.ca (E.W.); stefanie.schmitteckert@med.uni-heidelberg.de (S.S.); stefan.holland-cunz@ukbb.ch (S.H.-C.); constantin.schwab@med.uni-heidelberg.de (C.S.); patrick.guenther@med.uni-heidelberg.de (P.G.)

**Keywords:** Hirschsprung disease, calretinin

## Abstract

Introduction: Hirschsprung disease (HD) manifests as a developmental anomaly affecting the enteric nervous system, where there is an absence of ganglion cells in the lower part of the intestine. This deficiency leads to functional blockages within the intestines. HD is usually confirmed or ruled out through rectal biopsy. The identification of any ganglion cells through hematoxylin and eosin (H&E) staining rules out HD. If ganglion cells are absent, further staining with acetylcholine-esterase (AChE) histochemistry or calretinin immunohistochemistry (IHC) forms part of the standard procedure for determining a diagnosis of HD. In 2017, our Institute of Pathology at University Hospital of Heidelberg changed our HD diagnostic procedure from AChE histochemistry to calretinin IHC. In this paper, we report the impact of the diagnostic procedure change on surgical HD therapy procedures and on the clinical outcome of HD patients. Methods: We conducted a retrospective review of the diagnostic procedures, clinical data, and postoperative progress of 29 patients who underwent surgical treatment for HD in the Department of Pediatric Surgery, University of Heidelberg, between 2012 and 2021. The patient sample was divided into two groups, each covering a treatment period of 5 years. In 2012–2016, HD diagnosis was performed exclusively using AChE histochemistry (AChE group, n = 17). In 2017–2021, HD diagnosis was performed exclusively using calretinin IHC (CR group, n = 12). Results: There were no significant differences between the groups in sex distribution, weeks of gestation, birth weight, length of the aganglionic segment, or associated congenital anomalies. Almost half of the children in the AChE group, twice as many as in the CR group, required an enterostomy before transanal endorectal pull-through procedure (TERPT). In the AChE group, 4 patients (23.5%) required repeat bowel sampling to confirm the diagnosis. Compared to the AChE group, more children in the CR group suffered from constipation post TERPT. Discussion: Elevated AChE expression is linked to hypertrophied extrinsic cholinergic nerve fibers in the aganglionic segment in the majority of patients with HD. The manifestation of increased AChE expression develops over time. Therefore, in neonatal patients with HD, especially those in the first 3 weeks of life, an increase in AChE reaction is not detected. Calretinin IHC reliably identifies the presence or absence of ganglion cells and offers multiple benefits over AChE histochemistry. These include the ability to perform the test on paraffin-embedded tissue sections, a straightforward staining pattern, a clear binary interpretation (negative or positive), cost-effectiveness, and utility regardless of patient age. Conclusions: The ability of calretinin IHC to diagnose HD early and time-independently prevented repeated intestinal biopsies in our patient population and allowed us to perform a one-stage TERPT in the first months of life, reducing the number of enterostomies and restoring colonic continuity early. Patients undergoing transanal pull-through under the age of 3 months require a close follow-up to detect cases with bowel movement problems.

## 1. Introduction

Hirschsprung disease (HD) manifests as a developmental anomaly affecting the enteric nervous system, where there is an absence of ganglion cells in the lower part of the intestine. This deficiency leads to functional blockages within the intestines [1]. HD is usually confirmed or ruled out through rectal biopsy, a procedure that involves obtaining a minimum sample of 3 mm diameter from the rectal mucosa and the underlying submucosa [2]. Biopsies are performed at least 2 cm above the dentate line to avoid the physiologic aganglionic/hypoganglionic region in the distal rectum [3]. The identification of any ganglion cells through hematoxylin and eosin (H&E) staining rules out HD. However, if ganglion cells are absent, further staining with acetylcholine-esterase (AChE) histochemistry or calretinin immunohistochemistry (IHC) is routinely conducted as part of the diagnostic protocol for HD determination [3]. The advantages and disadvantages of these two staining methods in HD diagnostics have been discussed extensively in the literature in recent years [1,2,4,5,6,7]. What has not been described so far is the impact that the choice of staining method has on the course of curative surgical treatment of HD. In 2017, our Institute of Pathology at University Hospital of Heidelberg changed our HD diagnostic procedure from AChE histochemistry to calretinin IHC. In this paper, we report the impact of the diagnostic procedure change on surgical HD therapy procedures and on the clinical outcome of HD patients with a follow-up of 1 year after pull-through surgery.

## 2. Methods

**Figure 1:**
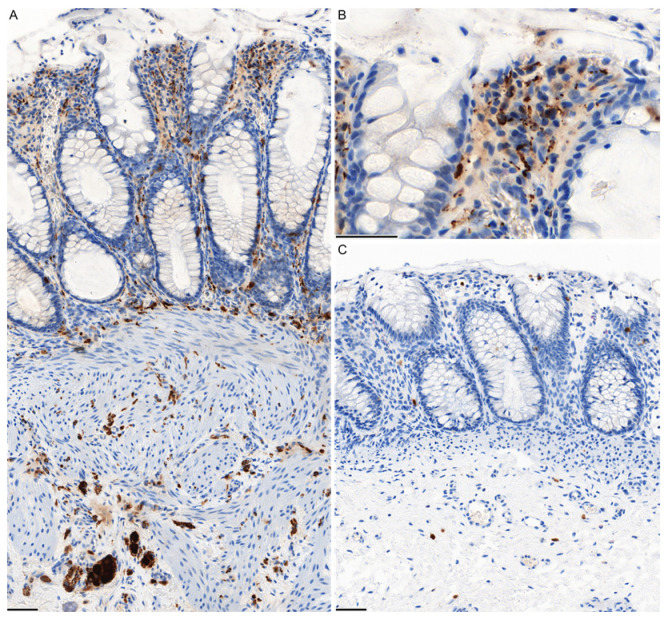
(**A**) Immunostain for calretinin with positively stained nerve fibers in the lamina propria, muscularis mucosae, and submucosa with ganglion cells in the left corner. (**B**) High-power magnification of the calretinin positive nerve fibers in the lamina propria. (**C**) In cases of HD, no staining in the lamina propria, muscularis mucosae, or submucosa is observed. Mast cells serve as an internal positive control. Bar equals 50 µm.

We conducted a retrospective analysis of the diagnostic methods, clinical records, and postoperative progress of 29 patients who underwent surgical treatment for HD in the Department of Pediatric Surgery, University of Heidelberg, between 2012 and 2021. Only patients who had symptoms typical of HD immediately postpartum and who had a positive HD diagnostic procedure performed in our clinic were included. The HD diagnosis was made in all cases by full wall rectal biopsies (2, 3, 5–6 cm above the dentate line) under general anesthesia. In addition, a contrast enema was used to visualize the change in colonic caliber. The patient sample was divided into two groups, each covering a treatment period of 5 years. In 2012–2016, HD diagnosis was performed exclusively using AChE histochemistry (AChE group, n = 17). In 2017–2021, HD diagnosis was performed exclusively using calretinin IHC (CR group, n = 12). The intestinal samples were processed by specialized pediatric pathologists at the University of Heidelberg. AChE histochemistry: Air-dried cryostatic sections were incubated for 1 h at 37 °C with an acetylcholinesterase staining kit (Bio-Optica, Milano, Italy, 30-50100, CND Code: W01030708) and washed with distilled water before mounting. Calretinin immunohistochemistry (Figure 1) was performed on an automated immunostainer (Ventana BenchMark Ultra, Ventana Medical Systems, Tucson, AZ, USA) using the biotin-free OptiView DAB IHC Detection Kit (Ventana Medical Systems, Oro Valley, AZ, USA). Then, 3 µm sections were cut from the formalin fixed and paraffin-embedded tissue blocks, deparaffinized, rehydrated, and pretreated with an antigen retrieval buffer (Tris/Borat/EDTA, pH 8.4). After blocking of endogenous peroxidase, the slides were incubated with monoclonal antibodies directed against calretinin (clone SP65, 6 µg/mL, Roche, Basel, Switzerland), followed by incubation with OptiView Universal Linker and OptiView HRP Multimer.

Transanal endorectal pull-through (TERPT), according to the De La Torre procedure, was performed as the therapeutic procedure for HD in all patients. For the TERPT procedure, transanal dissection was commenced 1 cm above the dentate line and a seromuscular cuff was used with a length of 1.5–2.0 cm. The length of the aganglionosis was determined by intraoperative whole-wall sectioning. Resection was performed at least 10 cm orally from the first detected ganglion cells. Four surgeons performed TERPT in the specified time interval.

The data extracted from patient records included patient demographics, presence of associated congenital anomalies, necessity for enterostomy before transanal endorectal pull-through (TERPT) and duration of enterostomy, age and weight at the time of rectal biopsy and TERPT, TERPT operative duration, length of aganglionosis, intestinal resection, and hospitalization period. We defined a long-segment HD as an aganglionosis of more than 30 cm. The reasons for placing an enterostomy included the occurrence of toxic megacolon, bowel perforations, or failure of bowel management by regular retrograde irrigation before TERPT. Additionally, postoperative complications were documented, with a follow-up period extending up to 12 months. These complications comprised anastomotic dehiscence, anorectal stenosis, ileus, enterocolitis, and constipation. Anorectal stenosis was defined as a narrowing of the coloanal anastomosis area necessitating transanal bougienage with Hegar dilators for a minimum of 10 days. The dilation treatment was carried out at home by the parents. Enterocolitis was defined by the occurrence of at least 3 out of 7 of the following symptoms: diarrhea (stool frequency higher than 3 times/day), fever, meteorism, vomiting, elevated infectious parameters on blood sampling (CRP/leukocytes), need for hospitalization, and administration of antibiotics. Constipation was rated by ROME III criteria [8]. Furthermore, we recorded stool frequency status and stool consistency one year after TERPT for all study participants. Stool consistency was assessed according to the Bristol stool chart (1 = hard lumps, 2 = lumpy sausage, 3 = sausage with surface cracks, 4 = smooth sausage, 5 = soft blobs, 6 = mushy stool, 7 = watery stool) [8]. Statistical analysis for all variables was performed using SPSS^®^ Version 26.0. The findings were presented as numbers, percentages, medians, means, and standard deviations. The two-sided Fisher’s exact test, independent samples t-test and Mann–Whitney U-test were employed to assess differences between the two groups, with statistical significance set at *p* < 0.05.

## 3. Results

Following the application of the inclusion criteria, the study enrolled 29 patients diagnosed with HD, with 17 patients in the AChE group and 12 patients in the CR group. Table 1 and Table 2 present demographic information, HD-related factors, perioperative details, and surgery-specific data. There were no notable discrepancies between the groups concerning sex distribution, gestational weeks, birth weight, or associated congenital anomalies. Although the AChE group had two children with complete aganglionosis (Table 1), and the mean length of the aganglionic segment was higher than in the CR group (AChE group 16.7 ± 15.1 cm vs. CR group 10.7 ± 7.5 cm) (Table 2), no statistically significant difference was observed. Almost half of the children in the AChE group, twice as many as in the CR group, required an enterostomy before TERPT (Table 1). Differences between groups in enterostomy duration were even more pronounced (AChE group 12.7 ± 14.5 months vs. CR group 2.0 ± 1.7 months) (Table 3). In the AChE group, there were 4/17 (23.5%) and in the CR group 2/12 (16.7%) patients with long-segment HD. All patients with long-segment HD required an enterostomy before TERPT. In comparison with the need for enterostomy in short-segment HD (AChE group 4/9, 30.8% vs. CR group 1/10, 10%), the need for enterostomy in long-segment HD was significantly higher in both groups (AChE group *p* = 0.029 vs. CR group *p* = 0.045).

In the AChE group, four patients (23.5%) required repeat bowel sampling to confirm the diagnosis. These four patients required an emergency exploratory laparotomy including enterostomy within the first 4 weeks of life. Rectal biopsies were indicated in all four of these patients for suspected HD, but initial biopsies could not yet confirm the diagnosis. In the CR group, all primary sampling was conclusive. Significant differences were seen between the groups in terms of age at rectal biopsy (AChE group 106.3 ± 65.3 days, range: 16–234 days vs. CR group 32.5 ± 25.5 days, range: 5–93 days) and TERPT (AChE group 252 ± 315 days, range: 20–1181 days vs. CR group 68 ± 33 days, range: 23–136 days). Relatedly, weight at TERPT differed between groups; patients in the CR group were younger at both rectal biopsy and TERPT, and, thus, lighter at TERPT, than patients in the AChE group (Table 2). No significant differences were observed between the groups regarding the duration of TERPT surgery, the extent of resection, or the duration of hospital stay post TERPT (Table 2).

No differences were seen between the groups in postoperative complications such as anastomotic dehiscence, anal stenosis, ileus, or enterocolitis (Table 4). Compared to the AChE group, more children in the CR group suffered from constipation post TERPT (Table 4). However, the duration of constipation symptoms was shorter in the CR group (mean 3.1 ± 2.7 months) than in the AChE group (mean 5.2 ± 3.4 months) (Table 3). CR group patients also required more intrasphincteric botulinum toxin treatments post TERPT and had lower stool frequencies one year after TERPT. However, none of these differences were statistically significant. Stool consistency one year after TERPT was similar between groups (Table 3).

## 4. Discussion

Hirschsprung’s disease is an anomaly characterized by the absence of myenteric and submucosal ganglion cells in the distal alimentary tract [9]. Histological diagnosis of HD presents pathologists with a considerable challenge and carries a great responsibility. If ganglion cells are not seen in H&E staining, additional staining with AChE histochemistry or calretinin IHC is standard procedure before determining a diagnosis of HD [3,10]. Increased AChE expression is associated with the hypertrophied extrinsic cholinergic nerve fibers of the aganglionic segment in most patients with HD [10]. This shows a pathomechanism typical for HD and is most commonly used [3]. However, AChE histochemistry is performed on frozen tissue and requires a quantitative and qualitative interpretation. If the intestinal tissue cannot be processed immediately by the pathologists, it should be frozen in liquid nitrogen and stored at minus 80 degrees Celsius. This treatment of the samples is more complex than formalin fixation, which is usually available in every operating theater. Thus, the success of the staining is greatly dependent on the experience of the pathologists and histotechnician [11]. The interpretation of the stain is associated with high rates of interobserver disagreement and false positives [12]. There is a lot at stake in the accurate interpretation of rectal biopsy findings, as a diagnosis of HD commits the patient to a major surgery. These factors can cause confusion for the clinician, require patients to undergo repeat biopsy procedures, and lead to delay in diagnosis and treatment [12]. Especially in neonates, the ganglion cells may be undifferentiated and difficult to identify [12]. The manifestation of increased AChE expression develops over time. Therefore, in neonatal patients with HD, especially those in the first 3 weeks of life, an increase in AChE reaction is not detected [7,10]. In the case of AChE, false negatives are predominantly associated with age, and the lack of an AChE reaction may not consistently rule out HD in very young neonates [3,10].

Calretinin, a calcium-binding protein dependent on vitamin D and found in both the central and peripheral nervous systems, has been investigated in the enteric nervous system as well. The lack of calretinin expression in HD was initially documented in resected specimens in 2004 [13], followed by observations on rectal mucosal biopsies [2,14]. Calretinin IHC identifies a particular group of enteric neurons along with their cell bodies and nerve fibers. In a normal ganglionic bowel, ganglion cells and nerve fibers that are calretinin-immunoreactive are easily discernible in the muscularis mucosae and lamina propria, respectively. Additionally, mast cells, which also exhibit calretinin expression, serve as a positive control for calretinin IHC [15]. Calretinin IHC reliably identifies the presence or absence of ganglion cells and offers multiple benefits over AChE histochemistry. These include the ability to perform the test on paraffin-embedded tissue sections, a straightforward staining pattern, a clear binary interpretation (negative or positive), cost-effectiveness [6], and utility regardless of the age of the patient.

In 2017, our Institute of Pathology at University Hospital of Heidelberg switched its HD diagnostics from AChE histochemistry to calretinin IHC. In this paper, we reported the impact on procedures of surgical HD therapy and clinical outcome of patients with a follow-up of 1 year after pull-through surgery. Our research comprised 29 patients who underwent surgical treatment for HD at the Department of Pediatric Surgery, University of Heidelberg, between 2012 and 2021; 17 received an HD diagnosis using AChE and 12 using calretinin IHC. Significant differences were seen between the groups in terms of age at rectal biopsy. In our sample, the mean time to HD diagnosis was 32 days (range of 1 to 93 days) in the CR group and 106 days (range of 16 to 234 days) in the AChE group. In the literature, the time to definitive HD diagnosis varies from 1 to 15 months [16]. Jorge et al. reported an average of 2.7 months (approximately 80 days) in their sample with only calretinin IHC diagnostics [1]. Singh et al. were able to diagnose 73% of all HD patients within the first 28 days of life using calretinin IHC [17]. Calretinin enables the diagnosis of HD to be made at any time. The ability to diagnose early allows pediatric surgeons to perform corrective operations earlier, reduces the need for enterostomy prior to TERPT, and prevents repeated intestinal biopsies until the diagnosis is made.

The longer the waiting time for HD diagnosis and TERPT, the higher the possibility of refractory obstructive symptoms. This is particularly important in patients with long-segment HD. In our sample, all patients with long-segment HD required an enterostomy before TERPT. In long-segment HD, conservative bowel management fails or remains a major challenge to prevent enterostomy. In our AChE group, almost twice as many enterostomies were necessary before TERPT compared to in the CR group (47.1% of patients vs. 25%). In addition, the average duration of enterostomy was significantly longer in the AChE group (12.7 months) compared to the CR group (2 months). One reason for this was the need for correction of associated heart defects in our patient population. In the CR group, 3/12 children (25%) were affected by congenital heart defects, and in the AChE group, 4/17 (23.5%) were affected. All four patients with heart defects in the AChE group, and two of the three patients with heart defects in the CR group, had an enterostomy. In contrast to the children in the CR group, all children in the AChE group had corrective surgery or cardiac catheterization before TERPT. In the case of a later HD diagnosis, children with congenital heart defects may experience a temporal overlap or competition of the necessary interventions. In this case, the interventions on the heart often take precedence, resulting in a further delay of the pull-through operation.

In the AChE group, four patients (23.5%) required repeat bowel sampling to confirm the diagnosis. These four patients initially underwent simultaneous rectal biopsy for exploratory laparotomy, including enterostomy, within the first 4 weeks of life. Due to the young age of the patients, the initial bowel samples were not conclusive. All second rectal samples showed HD with AChE histochemistry. In the CR group, all primary sampling was conclusive. Typically, pull-through surgery is carried out as an elective procedure within two months following diagnosis [3]. Thus, as time to diagnosis was significantly shorter in the CR group, we also observed a significant difference between groups in age and weight at the time of TERPT. Compared to a mean of 8.5 months and 6.8 kg in the AChE group, patients in the CR group were operated on 6 months younger, with a mean of 2.3 months and 4.4 kg.

Despite the age difference, there were no perioperative problems in the CR group. No significant differences between groups were seen in operative time or length of resection, aganglionosis, or hospital stay. With a follow-up of 1 year after TERPT, typical complications such as anorectal stenosis, enterocolitis, and constipation were observed in our groups with comparable incidence to previously published studies [18,19,20]. Wester et al. published data from HD patients who underwent surgery in the neonatal period. In their sample, the patients who underwent a one-stage procedure in the neonatal period showed the highest rate of anastomotic stenosis requiring peranal dilatation (40%) [19]. In our study, approximately 35% of the patients required short-term peranal dilatation treatments postoperatively. However, higher constipation rates and a larger number of intrasphincteric botulinum toxin treatments were noted in the younger CR group, though these results were nonsignificant.

One reason for the higher constipation rate in our younger CR group could be the still-lacking strength of the abdominal press. The standardized muscular cuff length used in the TERPT procedures (1.5–2 cm), which would have been longer for younger children relative to older children, could have also been a relative passage obstacle. In addition to retrograde irrigation, we treated these children early with intrasphincteric botulinum toxin. The success of this therapy was reflected in the difference in duration of constipation symptoms between groups. The mean duration of constipation symptoms within the first year after TERPT was shorter in the CR group (mean 3 months) compared to the AChE group (5.2 months) (Table 3). We also observed that the children in the AChE group had higher stool frequencies (22 bowel movements/week) one year after TERPT compared to the children in the CR group (14 bowel movements/week) (Table 3). This could be an indication that a TERPT with a one-stage procedure within the first 2–3 months of life helps infants in adapting bowel movements. Furthermore, establishing colonic continuity early in life may promote the development of normal continence [19]. Essentially, one-stage surgery for HD is firmly established, yielding outcomes that are comparable to or even superior to those achieved through two- or three-stage operations [19,21,22].

## 5. Limitations

This is a retrospective data analysis. It is fundamentally susceptible to interpretation errors. The validity of the statistical statement must be placed into perspective with a relatively small number of cases and without prospectively collected data. We are aware that due to the simultaneous care in the Department of Pediatric Cardiac Surgery at University Hospital Heidelberg, we treat a partially special cohort of Hirschsprung’s patients compared to other clinics.

## 6. Conclusions

Calretinin IHC enables the diagnosis of Hirschsprung’s disease to be made at any time, whether in premature infants, immediately postnatally, or after an emergency operation where the supply of fresh samples for AChE histochemistry can be significantly more difficult and formalin-fixation is more feasible. The ability of calretinin IHC to diagnose HD early and time-independently prevented repeated intestinal biopsies in our patient population and allowed us to perform one-stage TERPT in the first months of life, reducing the number of enterostomies and restoring colonic continuity early. Patients undergoing transanal pull-through under the age of 3 months require careful early follow-up to detect cases with bowel movement problems.

## Figures and Tables

**Table 1:** Hirschsprung associated and perioperative data.

	AChE Group	(n = 17)	CR Group	(n = 12)	*p* Value
	n	Percent (%)	n	Percent (%)	
Female	2	11.8	1	8.3	n.s.
Male	15	88.2	11	91.7	n.s.
Length of affected segment					
-Rectosigmoid	10	58.8	10	83.3	n.s.
-Colon	5	29.4	2	16.7	n.s.
-Jirásek–Zuelzer–Wilson	2	11.8	/	/	n.s.
Associated congenital anomalies	7	41.2	6	50	n.s.
Down syndrome	3	17.6	4	33.3	n.s.
Enterostomy prior to pull-through	8	47.1	3	25	n.s.
TERPT	12	70.6	10	83.3	n.s.
TERPT with laparotomy	3	17.6	2	16.7	n.s.
TERPT with laparoscopy	2	11.8	/	/	n.s.

TERPT = transanal endorectal pull-through; n.s. = not significant.

**Table 2:** Demographic, perioperative, and surgery-specific data.

	AChE Group	(n = 17)		CR Group	(n = 12)				*p* Value
	Mean	SD	Median	Mean	SD	Median	U	Z	
Weeks of pregnancy	38.8	1.4	39	38.0	1.5	38	76.5	−1.175	n.s.
Birth weight (g)	3329	572	3270	3114	669	2927.5	78	−1.062	n.s.
Age at rectal biopsy (days)	106.3	65.3	100	32.5	25.5	25	27	−3.321	0.000
Age at TERPT (days)	252	315	124	68	33	62	32.5	−3.077	0.001
Weight at TERPT (kg)	6.8	2.6	6	4.4	1.1	4.5	40.5	−2.725	0.005
Length of intestinal resection (cm)	31.6	14.7	27	23.3	6.2	21.5	69	−1.470	n.s
Length of aganglionic segment (cm)	16.7	15.1	8	10.7	7.5	8.5	84	−0.802	n.s.
TERPT operative time (min)	172.2	63.0	156	178.0	40.6	167.5	88	−0.621	n.s.
Length of hospital stay (days)	9.2	3.0	9	10.1	9.9	7.0	74	−1.252	n.s.

TERPT = transanal endorectal pull-through; n.s. = not significant; U = Mann–Whitney U-test; Z = Z-statistic.

**Table 3:** Clinical outcome data with a follow-up of 1 year postoperatively.

	AChE Group	(n = 17)			CR Group	(n = 12)					*p* Value
	n	Mean	SD	Median	n	Mean	SD	Median	U	Z	
Duration of constipation (month)	5	5.2	3.4	6	8	3.1	2.7	3	13.5	−0.976	n.s.
Bowel movements (per week)	17	22.8	17.9	20	12	14.5	9.5	14	72.5	−1.315	n.s.
Stool consistency *	17	4.8	0.9	5	12	4.5	0.5	5	77.0	−1.192	n.s.
Duration of enterostomy (month)	8	12.7	14.5	5	3	2.0	1.7	1	2.5	−1.990	0.048

* According to Bristol stool scale; n.s. = not significant; U = Mann–Whitney U-test; Z = Z-statistic.

**Table 4:** Postoperative complications.

	AChE Group	(n = 17)	CR Group	(n = 12)	*p* Value
	n	Percent (%)	n	Percent (%)	
Partial anastomotic dehiscence	1	5.9	/	/	n.s.
Anorectal stenosis	6	35.3	4	33.3	n.s.
Ileus	1	5.9	/	/	n.s.
Enterocolitis post TERPT	2	11.8	3	25	n.s.
Enterocolitis pre TERPT	1	5.9	1	8.3	n.s.
Constipation	5	29.4	8	66.7	n.s.
Sphinctary Botox treatment	6	35.3	7	58.3	n.s.

TERPT = transanal endorectal pull-through; Botox = botulinum toxin; n.s. = not significant.

## Data Availability

The data presented in this study are available on request from the corresponding author due to confidential patient data.
